# What sub-Saharan African countries can learn from malaria elimination in China

**DOI:** 10.1186/s41182-021-00379-z

**Published:** 2021-10-24

**Authors:** Abubakar Olaitan Badmos, Aishat Jumoke Alaran, Yusuff Adebayo Adebisi, Oumnia Bouaddi, Zainab Onibon, Adeniyi Dada, Xu Lin, Don Eliseo Lucero-Prisno

**Affiliations:** 1 National Primary Healthcare Development Agency, Abuja, Nigeria; 2 Global Health Focus, Kigali, Rwanda; 3 University Mohammad V, Rabat, Morocco; 4 University of Ilorin, Ilorin, Nigeria; 5 Ahmadu Bello University, Zaria, Nigeria; 6 Department of Thoracic Surgery, The First Affiliated Hospital, School of Medicine, Zhejiang University, Hangzhou, Zhejiang China; 7 Department of Global Health and Development, London School of Hygiene and Tropical Medicine, London, UK

**Keywords:** sub-Saharan Africa, Malaria, China, Elimination, Sustainable development goals

## Abstract

Malaria is one of the most devastating diseases plaguing the sub-Saharan African region since time immemorial. In spite of a global reduction in mortality rates, a significant proportion of deaths due to malaria is still accounted for in the region. China recently joined the 40 countries declared malaria free by the World Health Organization and became the first country in the WHO Western Pacific Region to be awarded the certification. We commented on the strategies employed by China to eliminate malaria, address challenges facing malaria control in sub-Saharan Africa, and derive lessons that could be learned in the sub-Saharan African context.

## To the editor:

Despite being preventable and curable and despite the concerted efforts geared towards it, malaria continues to remain one of the most worrisome vector-borne infectious disease plaguing the sub-Saharan Africa. In 2019, malaria accrued over 229 million cases globally; twenty-nine countries, sub-Saharan African region, accounted for the majority (95%) of these cases. Nigeria (27%), the Democratic Republic of the Congo (12%), Uganda (5%), Mozambique (4%) and Niger (3%) accounted for about 51% of all cases globally [1]. Despite a global reduction in mortality rates, a significant proportion of deaths—over 90% of 409,000 malaria deaths in 2019—is still accounted for in the sub-Saharan African region [1]. The most vulnerable population of people bearing the brunt and force of malaria are children under 5 and pregnant women. The United Nation Children and Emergency Fund asserts that a child under 5 dies of malaria every two minutes [2]. In 2019, over 12 million pregnancies in the WHO African region were exposed to malaria resulting in foetal loss, low birth weights and other morbidities [1, 3]. The WHO reported that the total funding for malaria control and elimination reached 3 billion dollars by 2019 with excess of 900 million dollars from governments of endemic countries [1, 4]. In spite of this, malaria remains largely uncontrolled in the sub-Saharan Africa region.

Recently, China joined the 40 countries declared malaria-free by the WHO, making it the first country in the WHO Western Pacific Region to be awarded the certification [5]. This notable feat followed a 70-year-long effort rife with strenuous but steady progress—from reporting 30 million cases annually in the 1940s to going 4 years without recording a single case [5]. In the wake of the coronavirus pandemic which has overburdened health systems globally, the cases of malaria in sub-Saharan Africa face an exponential growth [6]. This is due to interruption of malaria-control services, disruption of supply chains, and health facilities getting overwhelmed with towering cases of the coronavirus disease [7]. It therefore becomes imperative to give a new look to ways the sub-Saharan Africa region can continue its fight against malaria. Thus, in this paper, we commented on the strategies employed by China to eliminate malaria, address challenges facing malaria control in sub-Saharan Africa and derive lessons that could be learned in the sub-Saharan African context.

## Advances made in malaria control in SSA region

Malaria is a mosquito-borne disease, which in human, is caused by five protozoa: *Plasmodium falciparum*, *P. vivax*, *P. malariae*, *P. ovale*, and *P. knowlesi* [8]. Of all these, *P. falciparum* constitutes the single most important public health threat on a global scale, accounting for more than 90% of world’s malaria mortality [9]. It remains preponderant in Africa, largely as a result of optimal environmental condition for the female Anopheles mosquito vectors, amid sustained poverty [10]. Lack of full knowledge on its epidemic significance prevails, and subsequently it has been responsible for the enormous contribution to malaria burden of the world by the SSA region.

During early days to combat the debilitating effects of malaria in the 1940s and with the fresh discovery of chloroquine and dichlorodiphenyltrichloroethane (DDT), massive progress was attained and the incidence of malaria cases plummeted to considerable extents [9, 11]. However, by the 1960s, this ambition was arrested when it was realised that mass chemotherapy and residual spraying were laborious to sustain. And when this early attempts at elimination came to a forced, abrupt end, the resulting clinical and epidemiological consequences were brutal. Cases quickly returned to pre-intervention levels and in some cases with exacerbated aftermaths [9]. By 1990s, the cases escalated to epidemic proportions and this birthed the launch of new global strategy for elimination of malaria with particular focus on Africa. The most notable of these strategies is the Roll Back Malaria (RBM) initiative pioneered in 1998 by the WHO, UNICEF, UNDP and the World Bank in an effort to provide a coordinated global response to the disease [12]. The RBM offered fresh tools to combat malaria, exerting weights on six components including early detection of malaria illness; rapid treatment of those who are ill; multiple means for preventing infection; strengthening of health sector and intersectoral activities; a powerful sustained social involvement and movement; focused research for new tools and better implementation including rapid diagnostics; new drugs (artesunate) and new means for delivery; impregnated bed nets; new means for predicting epidemics (satellite mapping) [13].

The 2000s marked the years that saw the most increased financing for malaria control. Funding rose from an estimated 960 million dollars in 2005 to 2.5 billion dollars in 2014, depicting an unprecedented amount but still falling short of the estimated 5.1 billion dollars needed annually to attain international targets for malaria control and elimination [1]. This increased funding was accompanied by 40-fold increase in spending on Insecticide Treated Nets (ITNs), Rapid Diagnostic Tests (RDTs), Artemisinin-based Combination Therapies (ACTs) and Indoor Residual Spraying (IRS)—for vector controls, chemotherapies and chemoprevention—from 40 million dollars in 2004 to 1.6 billion dollars in 2014 [14]. 2015 was the target year set by the World Health Assembly and other institutions to reduce malaria incidence in countries that had malaria transmission in year 2000 by 75% [15]. And in line with reinforcing the efforts against malaria, when the Sustainable Development Goals were adopted in September of the same year, the objectives of reducing the burden of the disease were intrinsically linked to the items of the Sustainable Development Goals (SDGs) [16]. To reinvigorate progress, the WHO catalysed the “high burden to high impact” (HBHI) approach in 2018, together with the RBM Partnership to End Malaria. The response is led by 11 countries—including 10 in sub-Saharan Africa—that account for approximately 70% of the world’s malaria burden [1]. This new outlook focuses on drifting away from the one-size-fits-all approach to malaria control and instead choosing to implement tailored responses based on local data and intelligence.

These intensified efforts have yielded incredible results: globally, malaria deaths have reduced steadily over the period 2000–2019, from 736,000 in 2000 to 409,000 in 2019. The percentage of total malaria deaths among children aged under 5 years was 84% in 2000 and 67% in 2019 [1]. The global estimate of deaths in 2015, the Global Technical Strategy baseline, was about 453,000. The malaria mortality rate (i.e., deaths per 100,000 population at risk) reduced from about 25 in 2000 to 12 in 2015 and 10 in 2019, with the slowing of the rate of decline in the latter years [1]. Malaria deaths in the WHO African Region reduced by 44%, from 680,000 in 2000 to 386,000 in 2019, and the malaria mortality rate reduced by 67% over the same period, from 121 to 40 deaths per 100,000 population at risk [1].

Two important trends are manifest from the ongoing discussions: first, intense efforts translate to palpable results; second, in spite of progress made so far, the SSA region still live under conditions of holoendemic transmission. There is an increasing body of evidence to suggest the transmission belt of malaria is entrenched in middle Africa and incidence of new cases remains at disturbing levels, far from any inkling of elimination.

## Challenges facing malaria elimination in sub-Saharan Africa

Any understanding of malaria control in sub-Saharan Africa must take into account the challenges faced by the region. Foremost among these challenges is the predominant level of poverty amidst poor economic policies. Malaria remains a disease closely associated with poverty. Both a cause and consequence of poverty, malaria thrives mostly in regions with poor living conditions that foster the breeding of the mosquito vectors, and among people with poor socio-economic conditions that prevent them from accessing quality healthcare [17].

Secondly, the sub-Saharan Africa is located in a region with a climate suitable for the reproduction and propagation of the female anopheles mosquito. While the reason for the preponderance of malaria in this region is not limited to this, climactic conditions characterised by high temperature and abundant rainfall of sub-Saharan Africa play a major role [18]. In addition to this, there is a growing rate of mosquitoes’ resistance to the pyrethroids used in ITNs and parasites’ resistance to antimalarials, resulting in difficulty in curbing the disease in the sub-Saharan African region [19].

Thirdly, most countries in sub-Saharan Africa lack governmental support needed for effective control of malaria. In the absence of continuous and adequate financing of control programmes by the government and without judicious policies to safeguard against financial corruptive practices, most control strategies put in place invariably lose potency over time [20]. Additionally, the health system in sub-Saharan Africa is characterised by limited access to medical services and products, lack of human resources for health, shortage of functioning health facilities and overall lack of quality healthcare service delivery [20]. This results in poor malaria control and prevalence of malaria related morbidity and mortality in the region.

## Strategies of elimination of malaria in China

Before 1949, cases of malaria in China were as high as 30 million annually and deaths from this disease reached an annual number of more than 30,000 [21]. These ravaging statistics precipitated an integrated malaria control program that spanned generation and was unrelenting in its strategies. Consequently, the disease burden was forced into a sharp decline and epidemic areas shrunk at a remarkable rate. In 2017, for the first time, the country recorded zero indigenous cases of malaria—a feat that persisted for four consecutive years uninterrupted, and subsequently earned the country the malaria-free certification by the WHO [5, 21]. We look at the strategies employed by China in the elimination journey.

Foremostly, notwithstanding global efforts for malaria elimination, China, as a sovereign nation, ardently devoted resources and energy to finding a solution to the disease ravaging her citizens. This began as the 1967 effort that saw the launch of the 523 Project—a nation-wide research programme aimed at finding new treatments for malaria [5]. It was this project that yielded the discovery of ACTs, the most effective anti-malaria drugs available today. Long before the WHO recommended the use of ITNs, China was the first country to test their use in the 1980s and by 1988, over 2.4 million nets had been distributed nation-wide [5]. This action led to substantial reduction in the number of cases seen in the areas where the nets were deployed.

In 2010, China developed the National Malaria Eradication Programme (NMEP) and the Action Plan of China Malaria Elimination (ACPME), which was officially endorsed through the combined efforts of 13 ministries including the Ministries of Health, Finance, Education, Science and Technology and so on [22]. The main objective of the NMEP was to eliminate malaria by 2020 [22]. Concurrently, there was rapid socioeconomic growth and increasing urbanization that effect changes in the natural habitat of malaria vectors [23]. These efforts showed a dramatic curtailment of malaria cases. Only 3078 malaria cases were reported in 2014, among which only 56 were indigenous cases, a 98.6% enormous reduction from 4262 indigenous cases in 2010 [24]. The strategies employed include: effective blood tests for patients with fever using both active and passive tests, ensuring that over 33,182,304 people were tested and treated for malaria; widespread distribution of 3,201,493 Long-Lasting Insecticidal Nets (LLINs) and conduction of IRS with 857,701,275 individuals covered [24]. This translated to a dramatic reduction in the geographical distribution of malaria with 39 counties in Yunnan province reporting 397 indigenous cases of malaria in 2011 and only 1 in 2016 [25]. To maintain the progress made, the criteria for control and elimination of Malaria devised by the NMEP and the protocols developed by the National Health and Family Planning Commission of China were used to assess each county for reaching targets set for malaria elimination in order to heighten supportive supervision and surveillance [24, 26].

The success achieved is driven by several factors. Most importantly is the vehement political and financial commitment by the Chinese government. Up to 70 million dollars was doled out from central government finance and this continued despite the withdrawal of funding by The Global Fund in 2012 [24]. Secondly, the NMEP instituted strong regional collaboration with regions that have similar national and geographic conditions, consistent transmission intensities and epidemic factors [26]. This joint effort—overseen by different levels of government and health authorities—implemented same strategies through unified planning, monitoring and evaluation. Thirdly, data collection was reinforced through the Nation web-based case reporting system, which helped to answer relevant questions and evaluate outcomes [24]. And lastly, this development and implementation of the surveillance and response called “1-3-7” played immense role in prompt detection, treatment and response to individual cases of malaria, thus enabling tracing and elimination of infection sources as quickly as possible [27].

## Lessons sub-Saharan Africa can learn from China’s elimination of malaria

The current roadmap for eliminating malaria in WHO countries is the Global Technical Strategy for Malaria 2016–2030. It was adopted in May 2015 by the World Health Assembly and its aim is to provide technical guidance to countries and develop partners for the next 15 years through emphasizing the importance of scaling up malaria responses and moving towards elimination [15]. It also highlights the urgent need to increase investments across all interventions—including preventive measures, diagnostic testing, treatment and disease surveillance—as well as harness innovation and expand research [15]. The target of this strategy is to reduce global malaria burden by 90% [15]. While adopting this strategy, countries in the SSA can learn important lessons from China’s elimination story and co-opt the lessons therefrom to develop a culminated, scaled-up malaria response and intervention through preventive measures, diagnostic testing, treatment and disease surveillance.

On December 7–8, 2020, a 2-day symposium was convened by the National Institute of Parasitic Diseases in China in collaboration with WHO’s Global Malaria Programme and Harvard T.H Chan School of Public Health. The aim was to distil lessons that can be learned for malaria-endemic countries in Africa and emphasize China’s support to invest in academic partnerships and establish a China-African collaboration network [28]. This collaboration such as the China-Tanzania pilot project on malaria control that took place between 2015 and 2018 had huge impact on malaria control in Tanzania, leading to an 81% reduction in parasite prevalence in intervention regions [29]. Scaling up this type of cooperative intervention between Africa and China is therefore imperative. Some countries in the sub-Saharan African region such as Burkina Faso, Cameroon, Cote d’Ivoire, Sierra Leone, Tanzania, and Zambia have signed agreements with China to establish Institutional-based Networks of Cooperation between Africa and China on Malaria (INCAM) [21]. This communication platform will enable sustainable promotion of Africa–China cooperation to eliminate malaria. It will also provide a platform to share health products (e.g., quality control in production and delivery of antimalarial drugs), techniques (e.g., diagnostics) and intervention strategies [21]. This will furthermore promote south-south collaboration in the field of malaria control and elimination.

As with China, foremostly, SSA countries must emphasize government support. Malaria interventions are highly cost-effective and demonstrate one of the highest returns on investment in public health; however, the journey is long and tortuous and therefore support is likely to dwindle over time. Hence, governments should ensure that malaria-elimination programs are adequately and continuously funded; and they must devise strategies to get rid of corrupt practices that sabotage efforts through embezzlement and misappropriation.

Secondly, new operational strategies must be developed to achieve the goal set by the Global Technical Strategy for Malaria. For instance, network systems can be used to cover the swathe proportion of hard-to-reach population in the SSA regions. This will allow them to enjoy the dividends of elimination and control efforts as with urban areas. Additionally, there should be increased research efforts towards malaria including new diagnostic methods for cases of malaria with minimal parasitaemia, molecular techniques for identifying new strains, effective methods for monitoring drug resistance and vector susceptibility, and development and adoption of vaccines for malaria.

Third, it is no coincidence that malaria was eliminated in countries in China, and even Europe and United States of America at times when their economies saw exponential growths. Thus, the school of thought that proposed that Africa will have a breakthrough when it is lifted out of poverty proves accurate. Attempts must, therefore, be continued at ensuring development and implementation of rapid and sustained economic growth policies and programs such as education, nutrition and sanitation; individuals should be empowered and protected through social protection systems; contributors to social unrest such as ethnic and religious crises must be addressed to attain peaceful existence; and access to technology and innovations must be ensured.

Lastly, the health systems in sub-Saharan Africa must be strengthened; investments must be made to scale up intelligence and surveillance systems for appropriate decision making; there must be effective capacity building through training and retraining of healthcare workers to avoid health workforce attrition; the paucity of health facilities must be addressed through construction of modernised healthcare centres and through renovation of pre-existing, moribund ones; health financing must be invigorated to allow for equal access to healthcare regardless of individual’s financial status; intervention strategies must be targeted at communities through the Primary Healthcare system. Finally, the adoption of home-grown, country-specific solutions that are based on grassroots experience and that take into account the diversity across and within the region must be instituted.

## Conclusion

The recent elimination of Malaria in China has once again brought to the fore that even though the disease proves difficult to control, its elimination, and more so its eradication, is attainable through co-ordinated efforts and adoption of scientifically prudent means. Countries in sub-Saharan Africa must realize this and persevere in the fight against malaria by strengthening health systems, addressing emerging multi-drug and insecticide resistance and intensifying national, cross border and regional efforts to booster malaria response. So that in few years the huge burden of the disease borne by them will be reduced to infinitesimal level and the coveted malaria-free world we all dream of will be actualised.

## Data Availability

Data sharing not applicable to this article as no datasets were generated or analysed in the current study.

## Abbreviations

ACPME: Action plan of China malaria elimination
ACTs: Artemisinin-based combination therapies
DDT: Dichlorodiphenyltrichloroethane
HBHI: High burden high impact
IRS: Indoor residual spraying
ITNs: Insecticide treated nets
LLINs: Long-lasting insecticidal nets
NMEP: National Malaria Eradication Programme
RBM: Roll back malaria
RDTs: Rapid diagnostic tests
SDGs: Sustainable development goals
UNDP: United Nations Development Programme
UNICEF: United Nations International Children's Emergency Fund
WHO: World Health Organization
